# Temperature and socioeconomic vulnerability: associations with cardiac event-induced posttraumatic stress symptoms

**DOI:** 10.3389/fpsyg.2023.1092106

**Published:** 2023-06-01

**Authors:** Talea Cornelius, Joan A. Casey, Allan C. Just, Sebastian T. Rowland, Donald Edmondson

**Affiliations:** ^1^Center for Behavioral Cardiovascular Health, Columbia University Irving Medical Center, New York, NY, United States; ^2^Mailman School of Public Health, Columbia University Irving Medical Center, New York, NY, United States; ^3^Department of Environmental Medicine and Public Health, Icahn School of Medicine at Mount Sinai, New York, NY, United States

**Keywords:** climate change, temperature, socioeconomic status, posttraumatic stress disorder, acute coronary syndrome

## Abstract

**Background:**

Posttraumatic stress symptoms (PTSS) are common after acute coronary syndrome (ACS) and predict increased morbidity and mortality. Climate change contributes to worse mental and cardiovascular health outcomes, thus, PTSS represent a potential mechanism linking climate change to adverse cardiovascular outcomes. Because people living in areas with lower socioeconomic status (SES) experience greater climate vulnerability, have worse cardiovascular health, and may be more susceptible to PTSS, any effect of temperature on PTSS could be amplified in this population.

**Methods:**

Spatial regression models were estimated to test the association of temperature and temperature variability (within-day variability, directed change over time, and absolute change over time), census tract-level SES, and their interaction with PTSS 1 month post-hospital discharge in a longitudinal cohort study comprising 956 patients evaluated for ACS at an urban U.S. academic medical center between November 2013–May 2017. PTSS were self-reported in relation to the ACS event that brought the patient to the hospital. Census tract-level was computed as a composite score from the CDC Social Vulnerability Index, with higher values indicating lower SES.

**Results:**

No temperature or temperature variability metrics were associated with PTSS. Lower census tract-level SES was associated with greater PTSS at 1 month. There was a marginally significant interaction of SES with ACS status, such that we only observed evidence of an association among those with ACS.

**Conclusion:**

Temperature exposures were not associated with acute CVD-induced PTSS, which could be a result of a small sample size, mismatched timescale, or lack of a true effect. Conversely, lower census tract-level SES was associated with developing worse PTSS 1 month after evaluation for an ACS. This association appeared stronger in individuals with a true ACS. Early interventions to prevent PTSS could promote better mental and CVD outcomes in this at-risk population.

## Introduction

Climate change and its associated environmental stressors (e.g., increased frequency of heat waves, hurricanes, etc.) is a public health crisis (Watts et al., 2018). Without rapid, drastic reduction to global emissions, global heating is projected to exceed 1.5°C within 20 years and to surpass 2° within 30–40 years (Skea et al., 2022). Extreme heat events have already increased in frequency, and this trend is expected to continue (Intergovernmental Panel on Climate Change (IPCC), 2022). Given the evidence of the cardiovascular effects of temperature (Madrigano et al., 2013; Lavados et al., 2018; Li et al., 2018; Vered et al., 2020; Rowland et al., 2021) and the relationship between mental health and cardiovascular disease (Edmondson et al., 2012), incorporating temperature into the field of behavioral health is critical (Atwoli et al., 2021). Such research can help protect people, especially those experiencing cardiovascular disease such as acute coronary syndrome (ACS) from the impacts of climate change and inform health care in a rapidly-changing climatic regime.

Climate change-related temperature stressors have adverse implications for individuals with cardiovascular disease. In addition to associations of hotter temperatures with adverse outcomes such as incident ischemic stroke (Lavados et al., 2018; Li et al., 2018; Vered et al., 2020; Rowland et al., 2021) and risk for mortality after myocardial infarction (MI; Madrigano et al., 2013), warmer months and extreme heat events associated with a range of mental health outcomes, including more completed suicides (Galvão et al., 2018) and hospital admissions for schizophrenia (Shiloh et al., 2005), emergency psychiatric evaluation (Carlsen et al., 2019), and mania (Medici et al., 2016). ACS is already a highly distressing event, and posttraumatic stress symptoms (PTSS) after ACS are common (Edmondson et al., 2012). Because PTSS after ACS can double risk for adverse clinical outcomes (Edmondson et al., 2012), hotter temperatures may have additional, indirect associations with cardiovascular prognosis due to increased psychological distress. Finally, other temperature-related indicators of climate change also carry adverse implications for health, including associations of greater temperature variability with increased risk for incident MI (Rowland et al., 2021) and unusually cold weather with incident cardiovascular events (e.g., ischemic heart disease, hemorrhagic stroke; Madrigano et al., 2013; Li et al., 2018). Cold temperatures may influence adverse mental health outcomes as well (Carlsen et al., 2019), though this potential relationship is less established.

The impact of temperature stressors related to climate change on health is additionally distributed according to geographic differences in socioeconomic status (SES). Low SES is associated with greater disease burden, including cardiovascular conditions such as MI and stroke (Min et al., 2017), such that these populations are more susceptible to the health impacts of climate change-related stressors. Indeed, people with pre-existing cardiac conditions experience worse outcomes when exposed to extreme hot or cold temperatures (e.g., greater mortality risk for people with atrial fibrillation when exposed to extreme heat; Zanobetti et al., 2013). Some studies have also found that low SES is a risk factor for developing psychological distress, including PTSS, after large-scale disasters (e.g., earthquake and hurricane; Norris et al., 2002; Foa et al., 2006; Paxson et al., 2012; Hrabok et al., 2020) or following stroke (Bruggimann et al., 2006; Goldfinger et al., 2014), though research regarding the link between SES and PTSS broadly is more mixed. The exposure to warming, and the adverse health effects of warming, are more pronounced for individuals living in areas with lower SES (Zanobetti et al., 2013; Gronlund, 2014; Benmarhnia et al., 2015), and SES disparities in heat exposure are apparent even within small, urban geographical areas (Wong et al., 2016; Schinasi et al., 2018; Santamouris, 2020) like New York City (Rosenthal et al., 2014; Pioppi et al., 2020), an area where temperature increases have accelerated during recent decades (Horton et al., 2015; Storms, 2015) and heat waves have become more frequent (Depietri and McPhearson, 2018). Low SES additionally impedes an individual’s ability to cope with extreme temperatures (e.g., reduced access to air conditioning; Rosenthal et al., 2014; McDermott-Levy et al., 2021) and a review found that sleep disruption due to the effects of climate change – a known risk factor for adverse cardiovascular outcomes (Cappuccio et al., 2011), including re-hospitalization post-ACS (Romero et al., 2020) and PTSS following ACS and stroke (Rojas et al., 2018; Romero et al., 2020) – is most pronounced in low SES populations (Rifkin et al., 2018). Taken together, these findings suggest that low SES is both a multidimensional risk factor for exposure to extreme temperatures and a potential effect modifier, increasing exposure to climate change, reducing ability to cope with climate change, and increasing susceptibility to its effects due to pre-existing health disparities. Thus, the uneven distribution of morbidity and mortality secondary to a warming climate has great potential to further exacerbate health disparities.

In part due to a lack of large datasets that combine geospatial, medical, and psychosocial assessments, and despite the documented association between indicators of climate change and mental health, pathways relating temperature to cardiovascular health outcomes via psychological distress remain unexplored. Yet, psychological distress is a plausible mechanism that may explain the association of temperature with patient outcomes. Patients experiencing a cardiovascular event, such as ACS often develop posttraumatic stress symptoms (PTSS) secondary to the ACS experience (Edmondson et al., 2012). A recent meta-analysis found that as many as 13–20% of ACS patients develop clinically significant symptoms; this is non-trivial, because patients with elevated PTSS have doubled risk for cardiac event recurrence and mortality (Edmondson et al., 2012).

There is already theoretical support for a pathway from temperature to cardiovascular health via distress. The Enduring Somatic Threat model posits that attention to ongoing, internal cues, such as rapid heartrate when exercising, serve as ongoing reminders of the ACS, which can cause significant psychological distress (Edmondson, 2014). Exposure to extreme temperature triggers physiological symptoms like rapid heart rate (Ravanelli et al., 2015), which could potentiate greater somatic threat and increased risk for incident PTSS. Finally, while risk of PTSS appears to increase for those with low SES after large-scale disasters (e.g., earthquake; Norris et al., 2002; Foa et al., 2006); whether this extends to more subtle exposures, like temperature extremes, is uncertain.

The present study extends prior work on climate, SES, and cardiovascular health by asking whether climate vulnerability and socioeconomic vulnerability interact as causal effects (Hernán, 2018) of incident PTSS in patients evaluated for ACS. Utilizing a sample of patients evaluated for ACS at an urban academic medical center in New York City, we hypothesized the following:

*Hypothesis 1*: extreme temperatures (i.e., those that are increasingly hot and/or increasingly cold) will be associated with greater PTSS secondary to the ACS event.

*Hypothesis 2a–c*: greater temperature variability, including *(a)* within-day change (i.e., diurnal temperature range), *(b)* direction of change across days (i.e., mean first difference), and *(c)* overall variation between days (i.e., absolute mean first difference), further defined in the methods section below, will be associated with greater PTSS secondary to the ACS event.

*Hypothesis 3*: living in a census tract with lower SES will be associated with greater PTSS secondary to the ACS event.

Because people living in low SES areas may also be less equipped to deal with changes in temperature, we further hypothesized that:

*Hypothesis 4a–d*: lower census tract-level SES would confer greater susceptibility to temperature, such that greater associations of *(a)* extreme temperatures and *(c–d)* greater temperature variability with ACS-induced PTSS will be observed in individuals living in a lower SES census tract.

## Methods

### Participants

Participants were drawn from a large observational cohort study, REactions to Acute Care and Hospitalization (REACH; Birk et al., 2019). Eligibility criteria for REACH included *(1)* patients presenting to the NewYork-Presbyterian Hospital emergency department (ED) for evaluation for a suspected ACS (i.e., non-ST elevation myocardial infarction [NSTEMI] or unstable angina [UA]; patients with a diagnosis of suspected STEMI were excluded as they are typically taken immediately for cardiac catheterization, precluding the ability to participate in the baseline REACH interview; Anderson et al., 2007), *(2)* age 18 or older, and *(3)* English- or Spanish-speaking. Exclusion criteria were *(1)* non-cardiovascular terminal illness (life expectancy <1 year), *(2)* severe mental or psychiatric illness, *(3)* alcohol or substance abuse, and *(4)* unavailable for follow-up. For the present study, participants were required to live in one of the five New York City boroughs and provide complete data for analysis. These participants arrived in the ED and were recruited into REACH between November 2013–May 2017.

### Procedure

Potential participants were identified in the ED and approached by trained research assistants. After hearing an explanation of study procedures in either English or Spanish (per participant preference), interested participants provided written informed consent and completed a baseline demographic assessment and a brief psychosocial assessment. Medical information, including the Global Registry of Acute Coronary Events (GRACE) risk score (Granger et al., 2003; Eagle et al., 2004), Charlson comorbidity index (Charlson et al., 1994), and discharge diagnosis (suspected v. confirmed ACS) were extracted from the electronic health record. Approximately 3 days following baseline, participants completed a second psychosocial interview either while inpatient or at home via telephone where they reported baseline covariates (e.g., pre-existing PTSS and depression). A third interview occurred a median of 42 days post-discharge via telephone, during which participants reported PTSS specific to the suspected ACS. Sample size was determined to detect an association of probable ACS-induced PTSD diagnosis with ACS recurrence and mortality (Birk et al., 2019). This study was approved by the Columbia University Irving Medical Center Institutional Review Board, and all participants provided written informed consent before completing study procedures.

### Measures

#### Posttraumatic stress symptoms

PTSS keyed to the index ACS event, i.e., ACS-induced PTSS, were assessed at least 1 month after enrollment using the posttraumatic stress disorder (PTSD) Checklist specific to an acute stressor (PCL-S; Weathers et al., 1993). These items correspond to Diagnostic and Statistical Manual of Mental Disorders IV (DSM-IV) diagnostic criteria; this assessment was switched partway through the study to reflect the new diagnostic criteria in the DSM-V (PCL-5; Weathers et al., 2013). Scale items were selected and matched across the PCL-S and PCL-5 by psychologists with expertise in PTSD research to create a harmonized 17-item assessment (e.g., “In the past month, how much were you bothered by: […] Repeated, disturbing memories, thoughts, or images of the experience? […] Avoid thinking about or talking about the experience or avoid having feelings related to it?”). Response options ranged from 1 (not at all), to 5 (quite a bit), and responses were summed to create a total score, with a score ≥ 33 indicating probable PTSD (Weathers et al., 1993, 2013).

#### Temperature

Hourly temperature data on a 1 km^2^ grid resolution in New York City from 2013 to 2019 (Carrión et al., 2021) were obtained from a spatio-temporal model that leverages satellite and on-the-ground monitoring and integrated into the dataset. Mean temperature was calculated for each grid cell for each day.

#### Temperature variability

Using the hourly temperature data, we calculated three variability metrics. To capture within-day variability, diurnal temperature range was calculated as the difference between the day’s maximal and minimal hourly temperatures. Two aspects of variability across days were measured. Directed change over time, calculated as the difference between the current day’s mean temperature and that of the previous day, such that positive values indicate that temperature increasing over time (i.e., mean first difference), and overall change over time, calculated as the absolute value of that difference (i.e., absolute mean first difference; Rowland et al., 2021).

#### Socioeconomic status

Socioeconomic status at the census tract level was assessed using a composite score of four items from the CDC Social Vulnerability Index: percent below poverty, unemployment rate, *per capita* income, and percent aged 25 or older without a high school diploma. Possible scores range from 0 to 1, with higher scores indicating lower SES (i.e., greater vulnerability), and scores were computed to represent the years 2014–2018 (the most updated metric available in the dataset). These data were obtained from the Socio-Economic, Physical, Housing, Eviction, and Risk dataset (SEPHER; Tedesco et al., 2021) dataset.

#### Covariates

Demographic covariates included self-reported age, race/ethnicity, and gender. Medical covariates, including admission diagnosis (confirmed v. suspected ACS; i.e., the patient was determined to have a true ACS after enrollment into the study v. this diagnosis was not confirmed), Charlson comorbidity index (Charlson et al., 1994), and GRACE risk score (Granger et al., 2003; Eagle et al., 2004) were extracted from the medical record. Pre-existing psychological distress, including depression (Kroenke and Spitzer, 2002; Kroenke et al., 2009) and PTSS (Weathers et al., 1993, 2013), keyed to an event that occurred prior to the index ACS assessed via the Life Events Checklist (Gray et al., 2004), were self-reported at baseline.

### Data analysis strategy

#### Data harmonization

Temperature, census tract-level SES, and patient data were spatially joined using exact latitude and longitude of patients’ home addresses, assessed at baseline. Addresses were geocoded using the offline Geosupport Desktop Edition^™^ from nyc.gov (NYC Department of City Planning, 2021). Spelling errors were corrected in an iterative fashion to ensure address matching. Hourly temperature data were also matched to patient data at the day-level (e.g., based on date of hospitalization, date of assessment at the one-month interview). Mean temperature over the month (31 days) leading up to the assessment of PTSS was calculated by taking the mean of these 31 observations, censoring for days when the patient was still in the hospital; a similar procedure was followed for calculating temperature variability metrics over the 31 days preceding patient report of PTSS (i.e., each variability metric was calculated for each day, then averaged over the 31 day period).

#### Preliminary analyses

Geospatial data often necessitate statistical methods that can account for spatial dependence, i.e., more similarity in the outcome among participants living closer together. To assess the presence of spatial dependence, we first ran an ordinary least square regression predicting PTSS at 1 month, saved the residuals, and used these to compute Moran’s I test (Moran, 1950) to examine the significance of spatial dependence (a value of *p* < 0.05 indicates evidence for non-random error distributions). We additionally plotted a semi-variogram to examine the distribution of the residuals, and an upward trend, indicating more similarity between participants living closer together, an indicator of spatial dependence. We also mapped the spatial distribution of the residuals across New York City. These steps were followed for an unadjusted model, including SES and all temperature variables only, and then for a full model including SES, all temperature variables, and all covariates listed above.

#### Primary analyses

Spatial error models were estimated using the errorsarlm command in the spdep package (Bivand and Piras, 2015), implemented in R Version 4.1.2 (R 4.1.2, 2021; RStudio Version 1.4.1717). This model computes a linear association of predictor variables with the outcome variable, but errors are distributed according to a spatial autoregression. All models controlled for covariates listed above; unadjusted results are also reported. Significance of coefficients was set at *α* = 0.05. To test *Hypothesis 1*, we modeled the association between linear and squared mean daily temperature terms with PTSS, and we tested significance via the nested deviance comparison test with two degrees of freedom (one model). Mean temperature was centered at 70 degrees Fahrenheit and divided by 10, such that one unit change in mean temperature indicated a 10-degree difference, and squared mean temperature was equal to zero when the observed daily mean temperature was 70 degrees (for further context, if daily temperature was either 60 or 80 degrees [+ or –10 degrees], the squared term was equal to 1, at 50 or 90 this term was equal to 2, etc.).

To test *Hypothesis 2a–c*, we modeled associations for each of the temperature variability metrics (*a*: diurnal temperature range, *b*: mean first difference, and *c*: absolute mean first difference; three models total). To isolate the effect of variability alone, we adjusted for linear and squared daily mean temperature. Variability metrics are in degrees Fahrenheit and were not transformed (e.g., a diurnal temperature range of 5 is equivalent to a 5-degree Fahrenheit difference between the daily minimum and daily maximum temperatures, a mean first difference of 5 indicates that the current day is 5 degrees hotter on average than the previous day, and an absolute mean first difference indicates that the current day is either 5 degrees hotter or 5 degrees colder on average than the previous day). Only linear main effects were included for temperature variability, squared terms were not examined.

To test *Hypothesis 3*, we examined the association of census tract-level SES with ACS-induced PTSS across all four models described above. To test *Hypothesis 4a–d*, we included the interaction of census tract-level SES with mean temperature and temperature squared (*4a*) and each variability metric (*4b–d*) by adding the term(s) to each model above.

#### Sensitivity analyses

**Figure 1:**
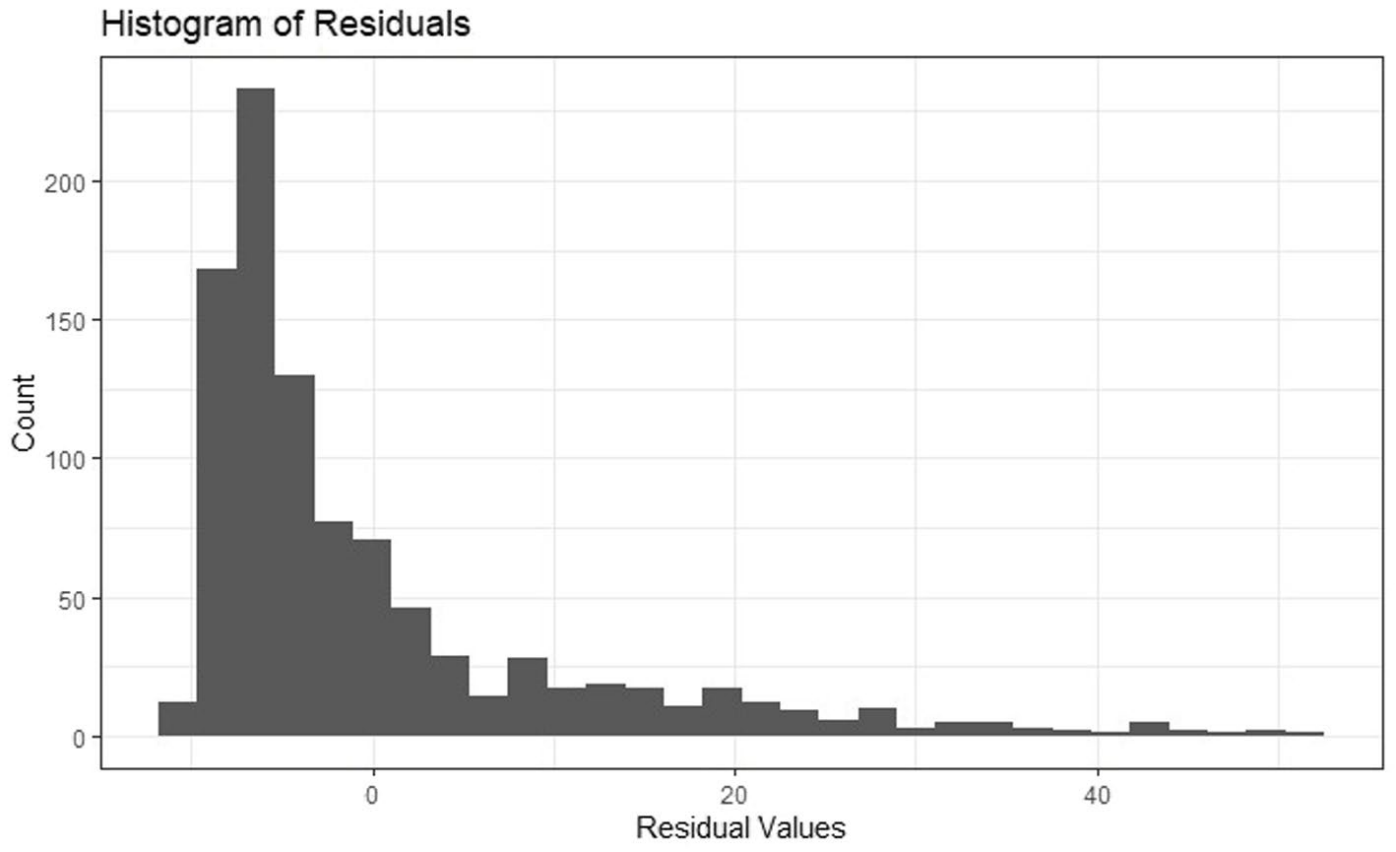
Histogram of residuals from ordinary least squares regression model predicting PTSS at 1 month from SES and temperature variables.

Residuals for linear models predicting PTSS were not normally distributed, as seen in Figure 1, and transforming PTSS did not remedy the situation. Thus, sensitivity analyses were conducted for *Hypotheses 1*–*3* using binary PTSS using the suggested clinical cutoff for a probable diagnosis of PTSD, with scores ≥33 = 1 and <33 = 0 (Weathers et al., 1993, 2013). This was accomplished by estimating general additive models with a spline smoothing over participant location (i.e., *X*- and *Y*-coordinates, which accounts for spatial dependence) from the mgcv package (Wood and Wood, 2015) in RStudio since, at the time of analysis, the errorsarlm model could not accommodate binary outcomes. Given the potential non-linearity of the association of SES with PTSS, we additionally conducted sensitivity analyses with SES as a categorical variable divided into quartiles.

#### Exploratory analyses

Reasoning that enduring threat may differ between participants with confirmed ACS (v. suspected ACS) as the index event, we tested exploratory interactions of SES and each temperature variable by index ACS status.

Data are not publicly available due to the inclusion of geospatial identifiers. Code is available from the first author upon request.

## Results

A total of 1,741 participants enrolled in REACH, 1,436 of whom provided valid addresses in New York City and were eligible for this study. Of these participants, 1,195 were matched by date and location to temperature data and 956 provided data for the present analysis (missing outcome, *n* = 54; *n* = 0; missing SES, *n* = 3; missing other [e.g., Charlson], *n* = 182). The median cardiac-induced PTSS score was 20.0 (*IQR* = 10.0; *Range* 17.0–76.0), and 17.7% of the sample met the clinical cutoff for a probable diagnosis of PTSD (score ≥ 33). Full demographic details are available in Table 1, correlations of temperature variables are in Table 2. Histograms for census tract-level SES, temperature variables, and PTSS at 1 month are in Supplementary Figures S1A–F.

**Table 1:** Demographic data for *N* = 956 participants.

		Mean (SD), median [IQR], or *n* (%)	Range
Age		61.06 (13.04)	21.82, 95.18
Race/Ethnicity	*White*	108 (11.3%)	
	*Black*	199 (20.8%)	
	*Other*	42 (4.4%)	
	*Hispanic*	607 (63.5%)	
Sex	*Female*	478 (50.0%)	
	*Male*	478 (50.0%)	
Borough	*Bronx*	251 (26.3%)	
	*Brooklyn*	37 (3.9%)	
	*Manhattan*	656 (68.6%)	
	*Queens*	12 (1.3%)	
Index ACS^a^	*Yes*	292 (30.5%)	
	*No*	664 (69.5%)	
GRACE^b^		93.49 (29.84)	23.00, 191.00
Charlson		1.00 [3.00]	0.00, 9.00
Baseline PTSS^c^		21.00 [17.00]	17.00, 85.00
	*Yes*	256 (26.8%)	
	*No*	700 (73.2%)	
Baseline depression		6.00 [9.00]	0.00, 24.00
PTSS^c^ at 1 month		20.00 [10.00]	16.95, 76.00
	*Yes*	169 (17.7%)	
	*No*	700 (82.3%)	
Mean temperature^d^		55.72 (17.15)	10.70, 88.17
Diurnal temperature range^d^		13.75 (1.84)	8.15, 19.25
Mean first difference^d^		0.01 (0.46)	−1.62, 1.30
Absolute mean first difference^d^		4.51 (1.55)	1.67, 8.39
Census tract-level SES		0.88 [0.26]	0.00, 1.00

a Acute coronary syndrome.

b Global registry of acute coronary events.

c Posttraumatic stress symptoms.

d Degrees Fahrenheit.

**Table 2:** Pearson correlations of temperature variables.

	Mean temperature	Diurnal temperature range	Mean first difference	Mean absolute difference
Mean temperature	1			
Diurnal temperature range	0.19	1		
Mean first difference	−0.31	0.34	1	
Mean absolute difference	−0.77	−0.12	0.04	1

### Preliminary analyses

**Figure 2:**
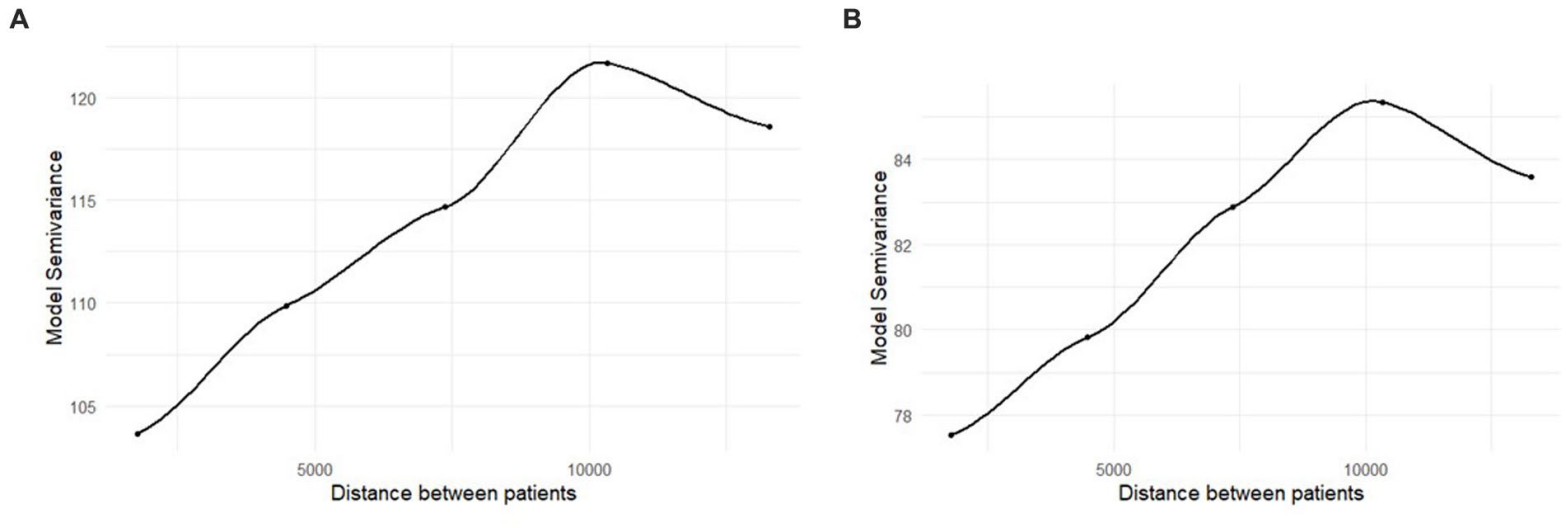
**(A,B)** Semivariogram of the residuals from the ordinary least squares regression models. Distance is in feet. The unadjusted results are in panel **(A)**, the fully adjusted results are in panel **(B)**.

Moran’s I statistic showed no evidence for residual spatial autocorrelation in either the unadjusted or the fully adjusted models, *p*s = 0.37 and 0.68, respectively. However, the semivariogram showed a distinct upward trend, suggesting greater similarity in residual values for points that are closer together spatially (i.e., there is less variance between observations that are spatially close relative to observations that are spatially distant; Figures 2A,B). Thus, we opted to continue with spatial regression models rather than reverting to ordinary least squares regression. Results from ordinary least squares regression models did not alter study conclusions.

### Primary analyses

Full results for *Hypothesis 1* and *2a*–*c* are in Table 3.

**Table 3:** Primary main associations estimated using spatial error models in the spdep package in R Version 4.1.2 (*N* = 956).

		Temperature only			Diurnal temperature range			Mean first difference			Absolute mean first difference		
		*B*	*se*	*p*	*B*	*se*	*p*	*B*	*se*	*p*	*B*	*se*	*p*
Intercept		10.15	2.19	<0.001	11.30	3.21	<0.001	9.97	2.19	<0.001	8.97	2.49	<0.001
Age		0.04	0.04	0.41	0.04	0.04	0.41	0.04	0.04	0.39	0.04	0.04	0.41
Race/Ethnicity	*Black*	0.72	1.24	0.56	0.67	1.24	0.59	0.65	1.24	0.60	0.73	1.24	0.56
	*Other*	0.67	1.75	0.70	0.67	1.75	0.70	0.67	1.75	0.70	0.72	1.75	0.68
	*Hispanic*	−0.53	1.13	0.64	−0.55	1.13	0.62	−0.60	1.13	0.60	−0.50	1.13	0.66
	*White*	*REF*	*REF*	*REF*	*REF*	*REF*	*REF*	*REF*	*REF*	*REF*	*REF*	*REF*	*REF*
Sex	*Female*	0.66	0.63	0.29	0.65	0.63	0.30	0.66	0.63	0.29	0.63	0.63	0.32
	*Male*	*REF*	*REF*	*REF*	*REF*	*REF*	*REF*	*REF*	*REF*	*REF*	*REF*	*REF*	*REF*
Index ACS^a^	*Yes*	1.98	0.67	0.003	1.95	0.68	0.004	1.93	0.68	0.004	1.97	0.67	0.004
	*No*	*REF*	*REF*	*REF*	*REF*	*REF*	*REF*	*REF*	*REF*	*REF*	*REF*	*REF*	*REF*
GRACE^b^		−0.03	0.02	0.17	−0.03	0.02	0.17	−0.03	0.02	0.16	−0.03	0.02	0.18
Charlson		0.39	0.21	0.060	0.39	0.21	0.06	0.39	0.21	0.056	0.38	0.21	0.069
PTSD^c^		0.28	0.03	<0.001	0.28	0.03	<0.001	0.28	0.03	<0.001	0.28	0.03	<0.001
Depression		0.43	0.06	<0.001	0.43	0.06	<0.001	0.43	0.06	<0.001	0.43	0.06	<0.001
Census tract-level SES		3.41	1.51	0.024	3.46	1.52	0.023	3.50	1.52	0.021	3.38	1.51	0.026
Temperature^d^		−0.32	0.39	0.40	−0.3	0.39	0.43	−0.34	0.39	0.38	0.00	0.50	1.00
Temperature squared^d^^,^^e^		−0.03	0.10	0.79	−0.03	0.10	0.79	−0.02	0.10	0.88	0.00	0.11	0.99
Temperature variability^e^					−0.08	0.17	0.63	−0.71	0.71	0.32	0.32	0.32	0.32
Lambda		0.01		0.75	0.01		0.79	0.01		0.84	0.01		0.84

a Acute coronary syndrome.

b Global registry of acute coronary events.

c Posttraumatic stress symptoms at baseline.

d Temperature was centered at 70 degrees Fahrenheit such that a one-unit change in mean temperature represents a change of 10 degrees Fahrenheit and temperature squared is equal to 0 when the mean temperature is 70 degrees.

e Calculated in degrees Fahrenheit.

*Hypothesis 1*: Neither mean temperature nor its squared term was significantly associated with PTSS, *B* = −0.32, *se* = 0.39, 95% CI −1.08, 0.43, *p* = 0.40, and, *B* = −0.03, *se* = 0.10, 95% CI −0.23, 0.18, *p* = 0.79, respectively. An omnibus test, computed as nested deviance comparison, confirmed that the temperature variables were not significantly associated with PTSS, *χ*^2^(2) = 1.72, *p* = 0.42. The unadjusted association of mean temperature and its squared term were not significantly associated with PTSS, *B* = −0.58, *se* = 0.46, 95% CI −1.48, 0.30, *p* = 0.20, and *B* = −0.07, *se* = 0.12, 95% CI −0.30, 0.17, *p* = 0.59, respectively.

*Hypothesis 2a*–*c*: Diurnal temperature range was not associated with PTSS related to the ACS, *B* = −0.08, *se* = 0.17, 95% CI −0.41, 0.25, *p* = 0.63. The associations of mean first difference, *B* = −0.71, *se* = 0.71, 95% CI −2.09, 0.68, *p* = 0.32, and absolute mean first difference, *B* = 0.32, *se* = 0.32, 95% CI −0.31, 0.95, *p* = 0.32, with PTSS were also not significant. In the three unadjusted models examining each temperature variability metric, no significant associations emerged (diurnal temperature range: *B* = −0.31, *se* = 0.20, 95% CI −0.70, 0.08, *p* 0.11; mean first difference: *B* = −0.83, *se* = 0.83, 95% CI −2.46, 0.80, *p* 0.32; absolute mean first difference: *B* = −0.02, *se* = 0.38, 95% CI −0.76, 0.72, *p* 0.96).

*Hypothesis 3*: In the model including mean temperature and temperature squared only, as socioeconomic vulnerability increased, PTSS related to the ACS increased significantly, *B* = 3.41, *se* = 1.51, 95% CI 0.44, 6.37, *p* = 0.024. Stated otherwise, a 1-unit change in census tract-level SES (from the lowest to the highest possible score) was associated with a 3.41-point increase in ACS-induced PTSS more than one month later. This effect remained significant in each of the three models including temperature variability, *p*s ≤ 0.026, and in the unadjusted models (for the model including mean temperature and temperature squared only, *B* = 3.63, *se* = 1.53, 95% CI 0.63, 6.63, *p* 0.018).

*Hypothesis 4a*–*d*: There was no interaction of census tract-level SES with temperature, *p* = 0.50, or temperature squared, *p* = 0.35 (omnibus, *χ*^2^(2) = 1.00, *p* = 0.61). Census tract-level SES also did not interact with diurnal temperature range, *p* = 0.31, mean first difference, *p* = 0.98, or absolute mean first difference, *p* = 0.83.

### Sensitivity analyses

In sensitivity analyses examining PTSS as a binary outcome, with a score ≥ 33 indicating probable PTSD, the main effect for the association of census tract-level SES with PTSS became marginally significant but remained positive. In the model with temperature and temperature squared only, this coefficient was, *OR* = 2.65, *p* = 0.076. This marginal effect replicated across the sensitivity analyses for models including temperature variability metrics.

In sensitivity analyses examining SES in quartiles, the main effect for the association of census tract-level SES with PTSS became marginally significant but remained positive, such that greater socioeconomic vulnerability was associated with endorsing more PTSS. In the model with temperature only, *χ*^2^(3) = 7.13, *p* = 0.068. Compared to those in the lowest quartile, those in the highest quartile (greatest vulnerability) had PTSS scores 1.78 points higher, *B* = 1.78, *se* = 0.95, 95% CI −0.08, 3.64, *p* = 0.061. For those in the second highest quartile (v. lowest quartile), *B* = 1.47, *se* = 0.89, 95% CI −0.27, 3.22, *p* = 0.098. This marginally significant effect replicated across the sensitivity analyses for models including temperature variability metrics.

### Exploratory analyses

In the model with temperature only, there was a marginal interaction such that census tract-level SES was associated with PTSS for participants with a confirmed ACS at the index event, *B* = 5.20, *se* = 2.82, 95% CI −0.32, 10.73, *p* = 0.065. For those without a true ACS at enrollment, the association of socioeconomic vulnerability with PTSS was, *B* = 1.59, *se* = 1.79, 95% CI −1.92, 5.10, *p* = 0.37. For those with confirmed ACS, *B* = 6.80, *se* = 2.39, 95% CI 2.11, 11.48, *p* = 0.004. This marginal interaction replicated across the sensitivity analyses for models including temperature variability metrics.

A marginally significant interaction emerged between confirmed v. suspected ACS and mean first difference, *B* = 2.45, *se* = 1.47, 95% CI −0.42, 5.32, *p* = 0.094. Simple slopes showed that, for those without a true ACS, mean first difference was marginally associated with lower PTSS, *B* = −1.42, *se* = 0.82, 95% CI −3.03, 0.20, *p* = 0.085. For those with confirmed ACS, this association was positive and not significant, *B* = 1.04, *se* = 1.25, 95% CI −1.32, 3.50, *p* = 0.41.

A marginally significant interaction emerged between ruling in for ACS and absolute mean first difference, *B* = −0.75, *se* = 0.43, 95% CI −1.59, 0.09, *p* = 0.080. Simple slopes showed that, for those without a true ACS, absolute mean first difference was not associated with PTSS, *B* = 0.55, *se* = 0.35, 95% CI −0.13, 1.24, *p* = 0.11. For those with confirmed ACS, this association was also not significant, *B* = −0.20, *se* = 0.44, 95% CI −1.05, 0.66, *p* = 0.65. No 3-way interactions between census tract-level SES, temperature or temperature variability, and ACS were statistically significant.

## Discussion

This study contributes to our understanding of the multifaceted impacts of climate change on health by testing potential associations of temperature and temperature variability with PTSS secondary to an ACS 1 month after ED evaluation. Results showed no evidence for the association of temperature with PTSS; however, living in a census tract with greater socioeconomic vulnerability was associated with significantly more PTSS symptoms.

Temperature extremes and variability resulting from the current climate crisis has increased cardiovascular risk for individuals with ACS (Madrigano et al., 2013; Lavados et al., 2018; Li et al., 2018; Vered et al., 2020; Rowland et al., 2021). Given the association of temperature with adverse mental health outcomes (Shiloh et al., 2005; Medici et al., 2016; Galvão et al., 2018; Carlsen et al., 2019) and cardiovascular symptoms (Ravanelli et al., 2015) that may serve as traumatic reminders of the ACS event (Edmondson, 2014), may also experience increased risk for PTSS secondary to the ACS. Because PTSS doubles risk for cardiac event recurrence and mortality (Edmondson et al., 2012), this may be one pathway through which individuals with ACS experience poor outcomes. Low SES may compound this risk, given geospatial inequities in the distribution of climate burden (Rosenthal et al., 2014; Wong et al., 2016; Schinasi et al., 2018; Pioppi et al., 2020; Santamouris, 2020; McDermott-Levy et al., 2021) and cardiovascular disease (Min et al., 2017), as well as a potential association of SES with PTSS following large-scale disasters (Norris et al., 2002; Foa et al., 2006).

The finding that census tract-level SES is associated with ACS-induced PTSS deserves greater attention. Literature linking SES to PTSS has been somewhat mixed and it can be hard to disentangle order effects (i.e., what came first – a diagnosis or lower SES?) (Koenen et al., 2017). This study disambiguates order effects by examining PTSS secondary to an acutely traumatic medical event at baseline enrollment; thus, all PTSS are necessarily newly acquired. It also shines a spotlight on health disparities by showing that, not only is socioeconomic vulnerability associated with a greater burden of cardiovascular disease (Min et al., 2017), but it also predicts the development of psychological trauma after cardiovascular events such as ACS. Thus, PTSS may be one mechanism of the association between low SES and adverse cardiovascular outcomes (Edmondson et al., 2012). Because exploratory interaction analyses suggested that this relationship is primarily true for patients with true ACS (v. those who had suspected ACS only), intervening early with individuals experiencing ACS who reside in areas with high socioeconomic vulnerability may be warranted to prevent psychological distress and its medical sequelae. A few promising interventions include those that utilize cognitive behavioral therapy or meaning making, but additional research is needed to confirm effectiveness and scalability (Birk et al., 2019). However, early intervention fails to address the root causes of socioeconomic disparities, and addressing disparities should be a priority.

Counter to our hypotheses, we did not observe association of temperature or any aspect of temperature variability with PTSS secondary to the ACS event; neither did interactions of temperature with census tract-level SES emerge. It is possible that there is simply no causal relationship between temperature and the development of these symptoms. However, it is also possible that the present study was unable to detect effects because they are small (and therefore the study may have been underpowered) or the timing of the assessments did not match the causal mechanisms as they unfold in real time. For example, the Enduring Somatic Threat model (Edmondson, 2014) would be better tested by examining the association of hotter days or hours with momentary symptoms and related intrusions (e.g., the somatic symptom of rapid heart rate during an extremely hot day (Ravanelli et al., 2015) might lead to heart-focused attention and intrusive, upsetting reminders of the ACS event). Future research should test this possibility. Indeed, even research on the association of temperature with event recurrence has highlighted the fine-grained associations of hourly temperature as a trigger for cardiovascular events such as stroke (Rowland et al., 2021). Other factors, such as changing locations or home air conditioning, may also have prevented the detection of temperature effects.

This study had numerous strengths, including integration of geospatial, medical, and psychosocial assessments to test novel and theory informed hypotheses with public health implications. PTSS is common after ACS and has significant adverse health consequences (Edmondson et al., 2012), so understanding the incidence of PTSS in this population has significant practical implications for improving patient health and well-being. There were also limitations. Participants were drawn from New York City and results may not generalize to other cities or to non-urban areas. Individuals living in other areas of the country may also be more vulnerable to other environmental stressors associated with climate change, such as catastrophic wildfires, with implications for both cardiovascular health (e.g., smoke exposure, and particulate matter concentration; Haikerwal et al., 2015; Reid et al., 2016; Jones et al., 2020; Chen et al., 2021) and PTSS (e.g., due to loss of life or property; Zhang et al., 2022). Incorporating these additional environmental stressors will be critical for conducting a more comprehensive assessment of the intersecting impacts of climate change and low SES for mental and physical health. Furthermore, although this study was completed prior to the COVID-19 pandemic, consideration of vulnerability and susceptibility to disease crises associated with both cardiovascular health (Li et al., 2020; Madjid et al., 2020; Richardson et al., 2020; Wu and McGoogan, 2020) and SES (Khanijahani et al., 2021) will be of interest in future work.

Given the marginal interactions of census tract-level SES and temperature variability metrics with ACS status, a larger sample comprising more patients with true ACS is also warranted. A larger sample size would also allow for an examination of potential effect modification by ACS type. Effect modification by other factors related to the ACS (e.g., delay to care receipt, in-hospital intervention) should also be tested in these samples, given that health disparities in ACS exist along the care continuum (Simon and Ho, 2020). Temperature data were keyed to participants’ home addresses, but participants likely spent time in other areas that may have had varying temperatures (e.g., walking in Central Park or along the river, running errands in midtown) and home temperatures, including access to fans and air conditioning, were not assessed. More detailed assessments of actual temperature exposure are warranted. Our ability to detect significant effects may also have been limited due to a lack of extreme temperatures within the dataset. For example, one would not expect extreme heat events to commonly occur during spring or fall months; similarly, variability during these months may be less problematic if all temperatures are relatively mild. Restricting the sample to only comprise participants who enrolled during summer or winter months would have severely limited the sample size, so this was not a viable option. It is also not clear that this would address the issue because temperature metrics were averaged over 31 days, such that a single extreme event would be less influential. Residuals in models predicting PTSS were not normally distributed, which can violate model assumptions in small samples. That said, results replicated across a wide range of sensitivity analyses, including models that treated PTSS as a binary score using an established cutoff for a suspected diagnosis of clinically significant PTSS. Finally, this is a prospective observational study, thus, estimates of causal effects may be confounded due to unmeasured factors. We are explicit about the goal of estimating causal effects to lend clarity to the conduct of our study and analyses (Hernán, 2018).

## Conclusion

The present study is among the first to link census tract-level SES to increased risk of developing PTSS secondary to evaluation for an acute cardiac event. Exploratory analyses suggested that this association occurs primarily among patients with confirmed ACS (v. those who ultimately did not receive an ACS diagnosis). Counter to hypotheses, temperature, and temperature variability were not associated with PTSS, nor did temperature variables appear to modify the relationship between SES and PTSS. These null findings may be due to imprecision in temperature estimates (e.g., not capturing in-home access to air conditioning, different locations throughout the day), a relatively small sample size, or a lack of any true causal effect, among other reasons. Because residing in low SES areas is associated with greater disease burden and increased morbidity and mortality, psychological interventions to prevent PTSS in at-risk patients experiencing ACS may be warranted. We should also prioritize protecting people residing in low SES areas from extreme temperatures and other climate change-related exposures.

## Data availability statement

The datasets presented in this article are not readily available because they contain geospatial information. Requests to access the datasets should be directed to tmc2184@cumc.columbia.edu.

## Ethics statement

The studies involving human participants were reviewed and approved by Columbia University Irving Medical Center Institutional Review Board. The patients/participants provided their written informed consent to participate in this study.

## Author contributions

TC: conceptualization, formal analysis, methodology, writing – original draft, and writing – review and editing. JC: methodology, supervision, and writing – review and editing. AJ: data curation, methodology, and writing – review and editing. SR: methodology and writing – review and editing. DE: funding acquisition, investigation, supervision, and writing – review and editing. All authors contributed to the article and approved the submitted version.

## Funding

This work was supported by the National Institutes of Health Science of Behavior Change Program through an award administered by the National Institute on Aging (https://www.nia.nih.gov/; U24AG052175; PI, DE). Cornelius receives support from the National Institutes of Health National Center for Advancing Translational Sciences (https://ncats.nih.gov/; KL2TR001874). Just receives support from the National Institute of Environmental Health Sciences (https://www.niehs.nih.gov/; P30ES023515 and R01ES031295; PI, AJ). Rowland receives support from the National Institute of Environmental Health Sciences (https://www.niehs.nih.gov/; R01ES028805). The content is solely the responsibility of the authors and does not necessarily represent the official view of the NIH.

## Conflict of interest

The authors declare that the research was conducted in the absence of any commercial or financial relationships that could be construed as a potential conflict of interest.

## Publisher’s note

All claims expressed in this article are solely those of the authors and do not necessarily represent those of their affiliated organizations, or those of the publisher, the editors and the reviewers. Any product that may be evaluated in this article, or claim that may be made by its manufacturer, is not guaranteed or endorsed by the publisher.
