# Supplementary material for: Temperature and socioeconomic vulnerability: associations with cardiac event-induced posttraumatic stress symptoms
Source: Front Psychol. 2023 Jun 1;14:1092106. doi: 10.3389/fpsyg.2023.1092106 (PMC10267367; doi:10.3389/fpsyg.2023.1092106)

**Supplementary Figure S1.** Histogram of raw census tract-level SES (**a)**, temperature and temperature variability in degrees Fahrenheit **(b – e)**, and PTSS at one month **(f)**.

**a.**
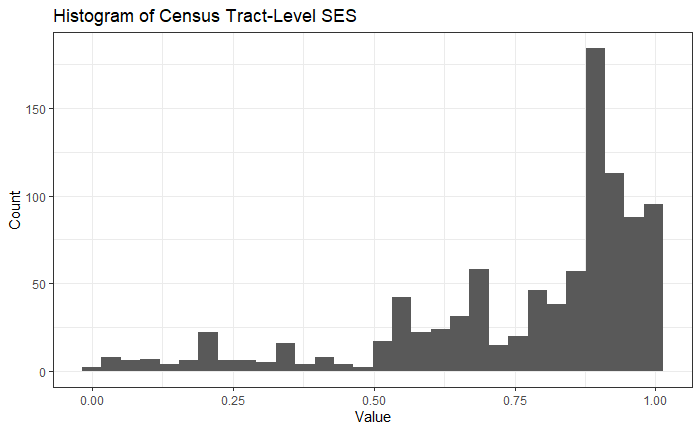
**b.**
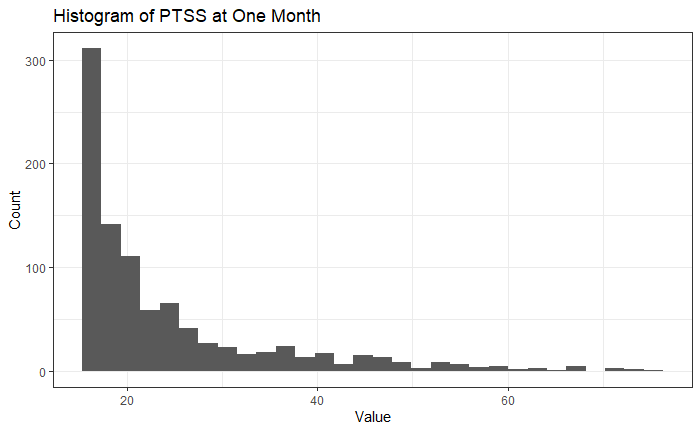


**c.**
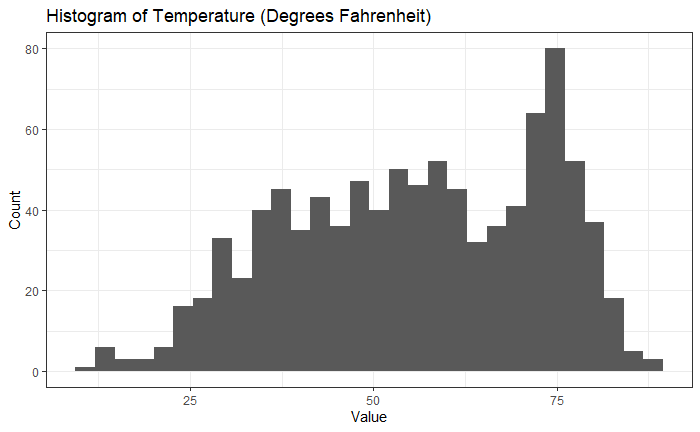
**d.**
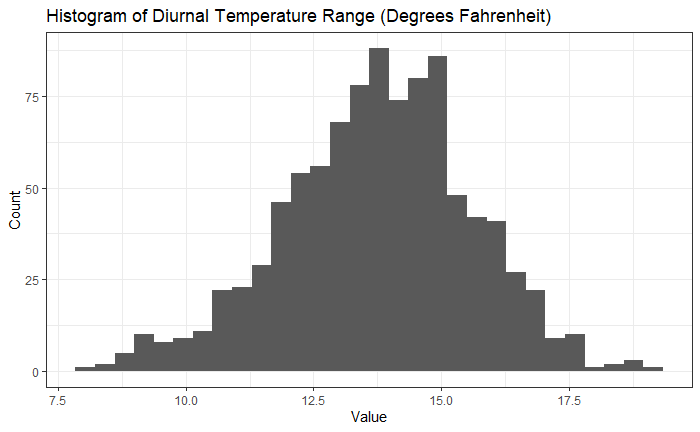


**e.**
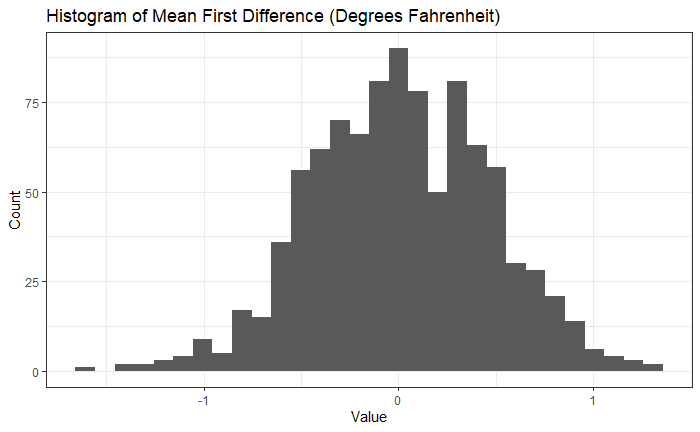
**f.**
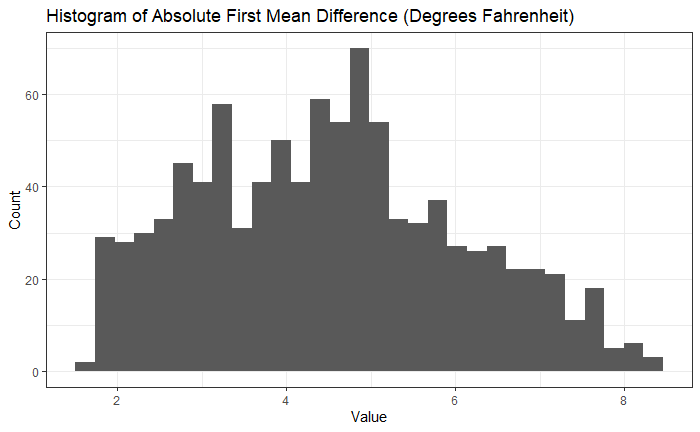

Supplement: Supplementary file 1 [file Table_1.DOCX]
